# Gen AI and research integrity: Where to now? The integration of Generative AI in the research process challenges well-established definitions of research integrity

**DOI:** 10.1038/s44319-025-00424-6

**Published:** 2025-03-24

**Authors:** Sonia Vasconcelos, Ana Marušić

**Affiliations:** 1 Science Education Program, Institute of Medical Biochemistry Leopoldo de Meis (IBqM), Federal University of Rio de Janeiro (UFRJ), Rio de Janeiro, Brazil; 2 Department of Research in Biomedicine and Health, University of Split School of Medicine, Split, Croatia

**Keywords:** History & Philosophy of Science, Science Policy & Publishing

## Abstract

The increasing use of Generative AI in the research process calls for a reassessment of research integrity and governance bases and puts the spotlight on the role of human agency in human-AI interactions.

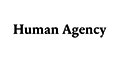

“[I]f you’re doing an experiment, you should report everything that you think might make it invalid—not only what you think is right about it: other causes that could possibly explain your results. Details that could throw doubt on your interpretation must be given, if you know them.” Richard Feynman gave this recommendation in his commencement address at Caltech in 1974, in which he explored what he called ‘Cargo Cult Science’ (Feynman, 1974). The difference between science and the cargo cult, Feynman explained, is “utter honesty”, which requires authors “leaning over backwards” to provide all relevant details including those that challenge their findings or interpretations. The goal would be to enable peers to form an accurate, unbiased understanding of the results, rather than one skewed in favor of a particular outcome desired by the authors.

The association between “leaning over backwards” in reporting results and scientific integrity aligns with and lends credence to Robert K. Merton’s “ethos” of science (Merton, 1973), by which the institutional imperatives of universalism, communality, disinterestedness and organized skepticism shape the research endeavor. The intriguing issue is that the culture reliant on such ethos does not necessarily reflect itself in the broader concept of research integrity that has been evolving over years. When Feynman described “cargo cult science” as lacking genuine scientific integrity, the prevailing notions of research integrity at that time failed to fully address the concerns, discourse and policies that would later be conceived in mainstream academia over the subsequent decades. In explaining “[w]hy must scientists become more ethically sensitive than they used to be”, John Ziman claimed that, for decades, there was a “no ethics” principle, essentially a taboo against discussing ethical considerations in research, which eventually became an outdated approach in scientific practice (Ziman, 1998). In the decades that followed Feynman’s considerations, doing science and talking about ethics became intertwined in the research culture—with disinterest and objectivity, for example, being tested against a more complex research endeavor connecting scientists across various institutions with distinct interests and commitments to knowledge production and governance.

The new research culture shaping post-academic science raised additional social and ethical responsibilities at the interface of science and society (Ziman, 1998). Over the decades, reflecting societal dynamics associated with the research endeavor, the concept of research integrity has expanded beyond Feynman’s approach focusing on individual researchers and their attitude toward doing and reporting science. While honesty is paramount and supported by transparency in conducting and reporting research, it also goes hand in hand with the promotion of good research practices. These practices include fair treatment of colleagues, research participants, the broader community, animals, and the environment, as well as responsible research procedures, safeguards for research and effective methods for reviewing and assessing science. Although all of these seem obvious and expected among researchers worldwide, the previously tacit ‘rules of the game’ for research and its outputs have evolved in such a way that research integrity became embedded in the public discourse of science. These more explicit rules have mirrored agreements over ethical issues across the research ecosystem, including universities, research institutions, and funding agencies, among other stakeholders (https://www.oecd.org/content/dam/oecd/en/publications/reports/2022/06/integrity-and-security-in-the-global-research-ecosystem_2bd8511d/1c416f43-en.pdf).

… reflecting societal dynamics associated with the research endeavor, the concept of research integrity has expanded beyond Feynman’s approach focusing on individual researchers and their attitude toward doing and reporting science.

## Research integrity in public discourse

During the past two decades, there has been a significant transformation in how academia addresses research integrity. A key aspect is the relationship between authors, the scientific article, and the larger research community. A notable illustration of these changes is the communication of preliminary results from neutrino experiments conducted by the Oscillation Project with Emulsion-tRacking Apparatus (OPERA). In September 2011, major scientific journals reported OPERA’s findings that a beam of neutrinos, sent 730 km through the Earth from Geneva, Switzerland, to the Gran Sasso laboratory in Italy, arrived approximately 60 nanoseconds earlier than expected. It sparked fascination but also skepticism among physicists given that the neutrinos seemed to have moved faster than the speed of light. The decision to submit the results to a scientific journal reflected the intrinsic complexities of scientific communication: while approximately 180 researchers backed the submission, 15 potential authors refrained from endorsing it (Cartlidge, 2011). In November 2011, despite replication of the experiment, growing skepticism in the scientific community continued to challenge the notion of “faster-than-light neutrinos” (Reich, 2011). Subsequent inquiries revealed a faulty fiber-optic connection and a miscalibration of the receiver’s clock (Reich, 2012). In March 2012, it was confirmed that neutrinos did not break the speed of light (Brumfiel, 2012). The InterAcademy Council’s 2012 policy report on responsible conduct in research highlighted that the “story illustrates that honest errors can occur in research, and that these can be corrected through subsequent work…” and that it “also raises the question of when and how research groups and institutions should announce or publicize results that would be considered revolutionary or anomalous.” (https://www.interacademies.org/publication/responsible-conduct-global-research-enterprise). The communication of OPERA findings and its scrutiny by peers shows a research landscape where scientific self-correction by authors should become the norm (Fanelli, 2016; Ribeiro et al, 2023).

In a nutshell, developing an open and balanced perspective on research integrity is a continuous process interconnected with the governance of research. Now, new elements are adding a layer of complexity to this endeavor: the rise of Generative Artificial Intelligence (Gen AI), with disruptive questions and transformative potential to challenge research governance and culture.

… developing an open and balanced perspective on research integrity is a continuous process interconnected with the governance of research.

## Changing the research culture with Gen AI

Gen AI is already affecting every stage of the research process from formulating a hypothesis to designing experiments, data analysis, visualization and interpretation of results, writing a research paper and even peer review (Ifargan et al, 2024; Binz et al, 2025; Naddaf, 2025). As it challenges both the notion of individual responsibility as well as community-standards of good research practices, integrating Gen AI into the research endeavor, while maintaining trustworthiness, has become an urgent demand in academia.

As described by Dua and Patel (2024), unlike most AI focused on pattern recognition, Gen AI can create new data it has never seen before. This new technology imposes a pressing need to revisit ethical standards and verification processes in research, including those related to the publication system. When it comes to experimental research, Gao et al (2024) envision AI scientist agents “as systems capable of skeptical learning and reasoning that empower biomedical research through collaborative agents that integrate AI models and biomedical tools with experimental platforms”, and note that intersections among technological, scientific, ethical, and regulatory domains are essential for governance frameworks. These concerns will become increasingly important as AI agents attain higher levels of autonomy (Gao et al, 2024).

AI research capabilities necessitate a robust and broader discussion in the research community on how AI systems can be aligned with the goals of maintaining integrity and trust in science. Discussions from an expert panel on ChatGPT and other Gen AI tools, convened in October 2023, suggest that a balanced approach is essential. The experts noted many benefits but also concerns about rapid dissemination of misinformation and extreme views, along with issues of copyright infringement and privacy among the panelists. (https://www.consilium.europa.eu/ro/documents-publications/library/library-blog/posts/panel-discussion-on-chatgpt-and-other-generative-ai-tools-risks-and-benefits/?filters=1492)

AI research capabilities necessitate a robust and broader discussion in the research community on how AI systems can be aligned with the goals of maintaining integrity and trust in science.

In “The Future of Human Agency”, Pew Research Center explored “how much control people will retain over essential decision-making as digital systems and AI spread” (2023, https://www.pewresearch.org/wp-content/uploads/sites/20/2023/02/PI_2023.02.24_The-Future-of-Human-Agency_FINAL.pdf). Pew and Elon University’s Imagining the Internet Center invited various stakeholders including David J. Krieger, Director of the Institute for Communication and Leadership in Lucerne, Switzerland. In Krieger’s vision “[i]ndividual agency is already a myth, and this will become increasingly obvious with time… Humanism attempts to preserve the myth of individual agency and enshrine it in law. Good design of socio-technical networks will need to be explicit about its post-humanist presuppositions in order to bring the issue into public debate. Humans will act in partnership—that is, distributed agency—with technologies of all kinds.” In the realm of scientific research, the concept of human agency has traditionally guided the integrity and rigor of inquiry and reporting. Especially with the advent of Gen AI, this framework is undergoing a profound transformation.

Researchers have started to navigate a rapidly changing environment regarding how they conceive, conduct, write, and evaluate research. A critical issue is reaching consensus among authors across different countries and fields, as we expand the understanding of human control in human-AI collaboration (Naddaf, 2025). While there is a strong foundation of research integrity established over decades, this process necessitates a reinvigorated dialog about the relationship between human agency and research integrity in this shifting landscape, as well as renewed definitions and guidelines that impact on practices in academia (Binz et al, 2025).

## A quadrant model of scientific research

**Figure 1:**
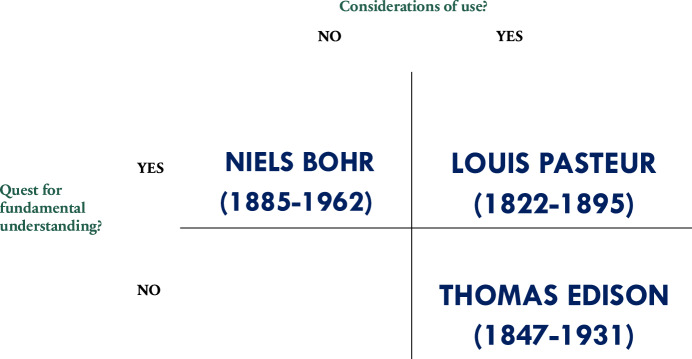
David Stokes’ quadrant model of scientific research. Adapted from Stokes (1997).

Donald Stokes developed the quadrant model, illustrating how research can simultaneously be driven by basic curiosity and a quest for practical applications (Stokes, 1997; Fig. 1).

**Figure 2:**
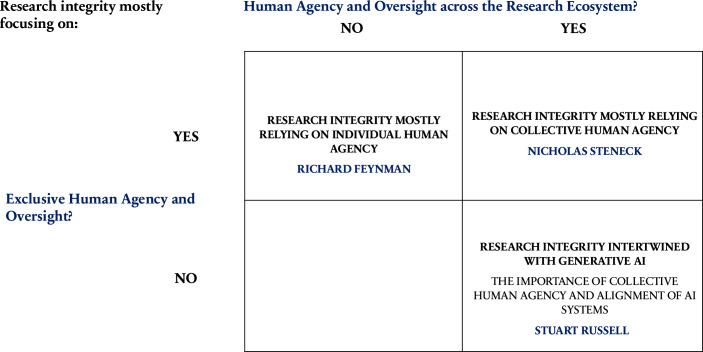
Quadrant Model of Gen AI and research integrity to conceptualize the interplay among research integrity, human agency, and Gen AI, inspired by Richard Feynman (1974), Nicholas Steneck (2006), and Stuart Russell (2019).

The upper-left quadrant represents Niels Bohr’s research, focusing on basic understanding and advancing knowledge, while the lower-right quadrant reflects Thomas Edison’s work with no direct interest in fundamental understanding. The upper-right quadrant, the famous Pasteur’s quadrant, illustrates the synergy between understanding and application—tackling real-word problems—and reflects Louis Pasteur’s legacy of application-inspired research in microbiology. In the lower-left quadrant, Stokes creates a space for knowledge production coming from research on “particular phenomena” without seeking general explanatory goals or immediate applications (Stokes, 1997). This area can include data collection, descriptive studies, or taxonomy, which may be vital for future theoretical or applied research but do not neatly align with either category. Inspired by “the tension between understanding and use” (Stokes, 1997), we propose a diagram to represent the tension between research integrity, human agency, and Gen AI (Fig. 2).

The upper-left quadrant illustrates Richard Feynman’s perspective on research integrity (Feynman, 1974), which prioritizes individual human agency unaffected by social incentives and reward structures. The upper-right quadrant reflects Nicholas Steneck’s broader approach to research integrity (Steneck, 2006), which relies both on individual scientists and on community principles and professional standards while noting the challenges scientists face in maintaining honesty and accountability within a complex research environment with different stakeholders. The lower-right quadrant is influenced by Stuart Russell’s concerns regarding AI alignment (Russell, 2019; Russell and Norvig, 2020). This quadrant recognizes that integrating research integrity with Gen AI requires enhanced human agency and oversight in a research process increasingly intertwined with Gen AI, requiring an expanded approach to and benchmarks for research integrity. Human agency and oversight are among the operational key requirements supporting ethical principles for AI systems established by the European Commission, as part of its “Responsible Use Generative AI in Research” (2024, https://research-and-innovation.ec.europa.eu/document/download/2b6cf7e5-36ac-41cb-aab5-0d32050143dc_en?filename=ec_rtd_ai-guidelines.pdf). Based on the “Ethics Guidelines for Trustworthy AI” (2019, https://digital-strategy.ec.europa.eu/en/library/ethics-guidelines-trustworthy-ai), the document presents three levels of human agency and oversight: human-in-the-loop (HITL), human-on-the-loop (HOTL), and human-in-command (HIC) approaches. HITL entails human intervention for the whole decision cycle; HOTL involves human input during the design cycle and continuous monitoring of the system’s operations; HIC encompasses human oversight of the entire activity of the AI system, including societal and ethical impacts, as well as decision-making for when and how to use the system in various contexts. The lower-left quadrant symbolizes the yet unclear and vague relationship between research integrity and, for example, human-AI collaboration. In this unnamed quadrant, we include AI research agents. As envisioned by Gao et al (2024) for biomedical fields, “rather than taking humans out of the discovery process” these AI agents can “combine human creativity and expertise with AI’s ability to analyze large datasets, navigate hypothesis spaces, and execute repetitive tasks…”. In this unnamed quadrant, we can have AI scientist agents potentially enhancing research integrity, and, at the same time, confronting notions of exclusive human agency and oversight in the research process.

## Human agency and issues of alignment in Gen AI interactions

An important and sensitive issue in reimagining research processes within human-AI collaboration is alignment: “the process of encoding human values and goals into AI models to make them as helpful, safe and reliable as possible” (https://www.ibm.com/think/topics/ai-alignment). Khamassi et al (2024) highlight timely questions concerning “strong and weak alignment” of Large Language Models (LLMs) with human values and the intricacies of AI’s understanding of these values. Apart from its caveats, alignment has a key role in fine-tuning LLMs to respond adequately to human intentions while mitigating harmful, toxic, or biased content (Ouyang et al, 2022). Open AI has introduced “*deliberative alignment*, a training paradigm that directly teaches reasoning LLMs the text of human-written and interpretable safety specifications and trains them to reason explicitly about these specifications before answering.” (https://openai.com/index/deliberative-alignment/).

Even if one regards LLMs as “stochastic parrots”, a term adopted by Bender et al (2021) to emphasize that these systems remix patterns in their training data without true understanding, *alignment* remains a critical problem. Given that training data is mostly derived from human-created data, it inherently reflects cultural patterns, worldviews, and social biases, along with the strengths and flaws of human knowledge production. As a result, Gen AI models risk reproducing—or even amplifying—biases in their outputs. When it comes to interacting with Gen AI models to formulate hypotheses, analyze data, or write a research report, how alignment shapes the output and behavior is a critical issue.

One question is how we can create a culture of research integrity that incorporates Gen AI while adhering to Feynman’s principles and preserving human agency over the process. Merely stating in the publication that AI systems or specifically LLMs were used to display data or to assist with the research report would not be sufficient; looking at the research process broadly, promoting research integrity should encourage more proactive discussions over human agency and alignment, particularly on how to produce reliable scientific content that will continue to feed into Gen AI models. When it comes to the research article, there are tons of data training LLMs, with new data constantly being produced within a publication culture that includes biased and persuasive reports with a focus on positive results.

One question is how we can create a culture of research integrity that incorporates Gen AI while adhering to Feynman’s principles and preserving human agency over the process.

Alignment concerns in the communication of science should encompass elements such as markers of excessive hype and exaggeration that have been present in research reports. Healthy rhetoric apart, ‘persuasive’ communication can potentially amplify an existing bias in research if we consider, for example, fine-tuning LLMs—and AI systems at large—with new data (Gao et al, 2024). Despite initiatives in the research community to make scientific reports more detailed, with including negative results or, in the clinical sciences, with the establishment of clinical trial registries that publish results from all trials, the existing body of training data is vast and spans a historical record affected by years of selective reporting and other issues.

Regarding Gen AI models, especially reasoning LLMs, they will continuously learn from the vast datasets of scientific communication in all fields. Whereas Gen AI with human-like introspection requires further studies and evidence to fully understand this attribute, reasoning LLMs should be an asset to the research process. In the clinical sciences, LLMs have already demonstrated promising performance in clinical reasoning in a study involving internal medicine residents and physicians at two medical centers in Boston (Cabral et al, 2024). In the communication of science, this human-AI collaboration incorporates various cultural and cognitive biases, the outcome of which is still an uncharted territory. All stakeholders in the research system have a responsibility to address this sensitive issue, and those dedicated to research integrity, communication, and policy should help with the exploration of the problem.

## A new paradigm for research integrity

The integration of Gen AI into research calls for a redefinition of research integrity. Reflecting on Feynman’s quadrant in Fig. 2, “leaning over backwards” to keep accurate reports should be the way forward. However, human-AI collaboration invites us to revisit the boundaries of collective human agency in the framework of Steneck’s research integrity (Steneck’s quadrant). We are heading towards Peter Levine’s approach to agency—another expert commenting in the Pew report: the ability of groups to deliberate and implement decisions. Whereas such collective agencies have already materialized among the research community, for example, with post-publication peer review in collective and open spaces such as PubPeer, and through stronger mechanisms to correct the research record, human-AI collaboration will gradually impact human agency in this endeavor. To cope with this problem, alignment has a key role to play, as illustrated in Russel’s quadrant.

Overall, research integrity entails adherence to ethical, transparent and rigorous scientific practices, relying on both individual and collective responsibility and a supportive research culture to maintain trust and credibility in research (Steneck, 2006). Although research integrity is framed in a way that assumes human agency as a given, the integration of Gen AI into the research process at all stages underscores the importance of emphasizing the role of ‘human agency’, with an explicit mention.

We propose that research integrity definitions should now incorporate “human agency”. This should remind us that Feynman’s principles of full honesty and Steneck’s approach to collective responsibility to adopt and promote responsible research practices are ultimately reliant on human agency, be it individual or collective. Research frameworks should now include strengthening human agency in proposing, conducting, communicating, and reviewing science. This expanded approach should also incorporate fostering research integrity benchmarks for training and deploying AI models and systems. In the biomedical sciences, these concerns are particularly relevant to the governance of AI agents. It is timely to address these sensitive issues in human-AI collaboration, given the growing capabilities of AI, which will lead to higher levels of influence in the research process. We advocate that academia should adopt a more proactive attitude toward seeking an understanding of the nuanced relationship between research integrity and human agency in times of profound transformation in knowledge production. This is not a long-term goal, as Gen AI has the potential to redefine patterns, reliability, and the overall culture of scientific communication.

We advocate that academia should adopt a more proactive attitude toward seeking an understanding of the nuanced relationship between research integrity and human agency…

## Supplementary information

Peer Review File
