# Supplementary material for: Gen AI and research integrity: Where to now? The integration of Generative AI in the research process challenges well-established definitions of research integrity
Source: EMBO Rep. 2025 Mar 24;26(8):1923–8. doi: 10.1038/s44319-025-00424-6 (PMC12018920; doi:10.1038/s44319-025-00424-6)
Supplement: Supplementary file 1 — Peer Review File [file 44319_2025_424_MOESM1_ESM.pdf]

EMBO reports

Where to go from here?

Sonia Vasconcelos and Ana Marušić

Corresponding author(s): Sonia Vasconcelos (svasconcelos@bioqmed.ufrj.br)

Review Timeline:

Submission Date:	5th Mar 25
Accepted:	11th Mar 25

Editor: Holger Breithaupt

Transaction Report: This article was editorially reviewed.
